# Emergence of a *Citrobacter amalonaticus* Strain Co-Producing Three Carbapenemases: Molecular Insights and Resistance Profiles

**DOI:** 10.14740/jmc5285

**Published:** 2026-03-27

**Authors:** Hawra Alsanna, Hussam Leskafi, Amani Alnimr, Shouq F. Alghannam, Sarah S. Alotaibi, Mohammad N. Alomary, Hatim Almutairi, Mashael Faisal Alghuraybi, Aisha Alamri

**Affiliations:** a Clinical Microbiology Department, Qatif Central Hospital, Al Qatif, Saudi Arabia; b Laboratory Medicine Department, King Fahad Hospital of the University, Al Khobar, Saudi Arabia; c Microbial Genomics Lab, Department of Microbiology, College of Medicine, Imam Abdulrahman Bin Faisal University, Dammam, Saudi Arabia; d Applied Genomic Technologies Institute, King Abdulaziz City for Science and Technology (KACST), Riyadh 11442, Saudi Arabia; e National Livestock and Fisheries Development Program (NLFDP), Riyadh, Saudi Arabia; f Department of Clinical Laboratory Sciences, College of Applied Medical Sciences, Imam Abdulrahman Bin Faisal University, Dammam, Saudi Arabia

**Keywords:** *Citrobacter amalonaticus*, OXA-48, NDM, KPC, Carbapenemase, Whole-genome sequencing

## Abstract

The emergence of carbapenemase-producing organisms poses an escalating public health risk. In this case study, we used whole-genome sequencing (WGS) to characterize the carbapenem resistance mechanisms of a multidrug-resistant *Citrobacter amalonaticus* (*C. amalonaticus*) strain recovered from a surgical site infection. The isolate was initially identified using a MALDI-TOF mass spectrometry system. Phenotypic antibiotic susceptibility testing revealed the presence of carbapenem-resistant *C. amalonaticus* isolate with high-level resistance to both imipenem and meropenem (imipenem minimum inhibitory concentration (MIC) ≥ 32 mg/L; meropenem MIC ≥ 32 mg/L). Rapid screening of the carbapenemase-encoding genes using the GeneXpert system revealed the presence of *bla*OXA-48, *bla*NDM, and *bla*KPC. WGS performed on an Illumina NovaSeq 6000 platform demonstrated the presence of *bla*NDM-1, which was chromosomally integrated. *In silico* analysis suggested that *bla*KPC-2 and *bla*OXA-48 were associated with distinct plasmid replicons (IncQ2 and IncL/M, respectively), although complete plasmid reconstruction was not possible using short-read sequencing. The convergence of multiple carbapenemase genes in *C. amalonaticus* indicates an urgent shift in the landscape of carbapenem resistance beyond that of traditional high-risk pathogens. These findings underscore the need for enhanced active surveillance of *Citrobacter* spp. as emergent reservoirs of carbapenemase genes. Early detection of these novel resistance determinants is essential for strengthening targeted infection control strategies and optimizing antimicrobial stewardship interventions at regional, national, and global levels.

## Introduction

Antimicrobial resistance (AMR) remains a major global concern, and prolonged exposure to antibiotics drives multidrug-resistant (MDR) infections, particularly in high-risk groups [1]. The emergence and dissemination of carbapenem-resistant pathogens are recognized as a major concern by the World Health Organization (WHO), which has ranked carbapenem-resistant *Enterobacterales* (CRE) as “critical priority pathogen’’ requiring urgent clinical attention [2]. In Saudi Arabia, pooled carbapenem resistance among *Enterobacterales* ranged from 6.6% to 18.6% [3]. However, carbapenemase production remains less common in *Citrobacter* spp. than in *Klebsiella pneumoniae* or *Escherichia coli* (*E. coli*), although emerging reports indicate rising resistance levels [4]. This emphasizes the importance of collaborative efforts and active surveillance to control the spread of these difficult-to-treat pathogens.

This report describes the genomic characterization of a carbapenem-resistant *Citrobacter amalonaticus* (*C. amalonaticus*) isolate co-harboring three carbapenemase genes (*bla*NDM-1, *bla*KPC-2, and *bla*OXA-48), recovered from a surgical site infection (SSI) in Eastern Saudi Arabia. To our knowledge, this is the first documented case of triple carbapenemase coproduction in *C. amalonaticus* in this region. The objectives of this case report are to define the complete resistance profile and genomic architecture of this isolate using whole-genome sequencing (WGS), and to contextualize this finding within the regional epidemiology of carbapenem resistance, highlighting the emerging role of *Citrobacter* species as potential reservoirs for high-risk carbapenemase genes.

The study was conducted in accordance with the ethical principles outlined in the Declaration of Helsinki by the World Medical Association. Ethical approval was obtained from the Institutional Review Board (IRB) at Qatif Central Hospital (Research Bioethics Committee) (Approval No. QCH-SRECO 15/2025).

## Case Report

A 24-year-old male of Pakistani origin who had resided in Saudi Arabia for several years prior to admission and worked as a carpenter sustained a severe occupational injury from an electrical saw, resulting in extensive trauma to the proximal thigh involving the skin, subcutaneous tissue, muscle, femur, and major blood vessels. He was admitted in a hemodynamically unstable condition and underwent emergency vascular and multidisciplinary surgery to preserve the limb. Despite the initial salvage, progressive gangrene developed, necessitating amputation. The postoperative course was complicated by recurrent, unresolved wound infections caused by multiple organisms, the most recent of which was *C. amalonaticus*. This isolate was found to be carbapenem- and extensively drug-resistant; phenotypic testing demonstrated resistance to nearly all tested agents. Colistin remained reliably active (minimum inhibitory concentration (MIC) 0.94 mg/L), while amikacin, trimethoprim–sulfamethoxazole, nitrofurantoin, and fosfomycin retained activity in confirmatory testing. The isolate was subsequently used for advanced molecular characterization, including WGS.

### Material and methods

#### Bacterial strain

*C. amalonaticus* was isolated from an SSI in the patient. The isolate was identified using the VITEK 2 and VITEK MS systems.

#### Antimicrobial susceptibility testing

Initial testing of the antibiotic sensitivity of the isolate was performed using the VITEK 2 system. The isolate was resistant to Augmentin, cefazolin, cefuroxime, ceftriaxone, ceftazidime, cefepime, piperacillin-tazobactam, meropenem, imipenem; intermediate to gentamicin; and sensitive to amikacin, trimethoprim-sulfamethoxazole, nitrofurantoin, and fosfomycin.

Further antimicrobial susceptibility testing was performed to determine the MICs of the supplemental agents. Results were as follows: colistin E-test MIC 0.94 mg/L (sensitive), ceftazidime-avibactam E-test MIC ≥ 256 mg/L (resistant), meropenem E-test MIC ≥ 32 mg/L (resistant), and imipenem E-test MIC ≥ 32 mg/L (resistant), aztreonam disc was resistant.

#### Molecular testing of carbapenemase-producing genes

This was carried out using the GeneXpert system (Cepheid, USA), which showed positive results for the NDM, KPC, and OXA-48 genes.

#### DNA extraction

An overnight culture of the *Citrobacter* strain was used for DNA extraction using the QIAamp DNA Kit (Qiagen, Germany) following the manufacturer’s recommendations.

#### Library preparation and WGS

DNA concentration was measured using a Qubit Fluorometer 2.0 to ensure accurate quantification before library preparation, using the Illumina DNA Prep kit (Illumina, USA). A total of 450 ng of genomic DNA was extracted from each sample. The DNA was fragmented using Taq buffer 1 (TB1) and tagged using bead-linked transposomes (BLT), followed by termination of the reaction using Tagment Stop Buffer. The samples were purified using Tagment Wash Buffer to remove impurities. The libraries were amplified using PCR with Enhanced PCR Mix, incorporating i5 and i7 index adapters to ensure compatibility with low-plexity pooling and maintain color balance. The amplified products were purified using Sample Purification Beads and washed using freshly prepared 80% ethanol solution. Purified libraries were resuspended in Resuspension Buffer. The library size distribution was evaluated using the LabChip® GX II Touch 24 system (Revvity, USA), which demonstrated an average fragment size of approximately 600 base pairs (bp).

#### Sequencing preparation and sample run on NovaSeq 6000

WGS was performed using the Illumina short-read technology, which generates high-quality paired-end reads suitable for accurate base calling, variant detection, and AMR gene identification. Libraries were pooled and adjusted to 2.7 nM as a pre-sequencing normalization step before the final dilution for loading. The denaturation process was carried out using 0.2 N sodium hydroxide (NaOH), followed by neutralization with 400 mM Tris-HCl according to the Illumina Denaturation and Neutralization Protocol. The prepared libraries were loaded onto an S2 Flow Cell (200-cycle kit) and run on a NovaSeq 6000 platform according to the manufacturer’s specifications.

#### Bioinformatics analysis and sequence alignment

The sequence file was subjected to comprehensive *de novo* genomic analysis using the Bactopia pipeline v3.2.0[5], which is built upon the nf-core framework for reproducible and scalable workflows [6]. This pipeline integrates multiple open-source tools and databases [5] to carry out quality control, genome assembly, annotation, typing, and resistance profiling.

The workflow began with raw sequencing of short reads that were quality-filtered and assembled using SPAdes v3.15.5 [7]. This step reconstructs the bacterial genome without relying on a reference, thereby enabling the study of novel or uncharacterized isolates.

Assembly quality was evaluated using contig number, total genome length, N50, L50, and GC content to indicate reasonable completeness. Gene prediction and functional annotation were performed using Prokka v1.14.6 [8] to identify both coding and non-coding genomic features. Taxonomic classification was performed using two approaches to confirm organism identity. Mash v2.3 [9] compared the assembled genome with the National Center for Biotechnology Information Reference Sequence (NCBI RefSeq) database and identified the most similar known strains. Sourmash [10] was used with the Genome Taxonomy Database (GTDB) [11] to assign standardized and phylogenetically informed taxa. Both tools provide strain-level similarities and species-level evolutionary contexts.

Strain typing was performed using multilocus sequence typing (MLST) v2.23.0, with the *Citrobacter freundii* scheme from the PubMLST database [12]. This approach detects allele variants in housekeeping genes to determine an isolate’s sequence type (ST), which supports epidemiological tracking and comparison across studies.

AMR and stress response genes were identified using ABRicate v1.0.1 [13], which screens genomic assemblies against curated resistance gene databases. The screened databases included the Comprehensive Antibiotic Resistance Database (CARD) [14], ResFinder [15], ARG-ANNOT [16], NCBI AMRFinderPlus [17], and BacMet [18]. Genes were reported when the alignment identity and coverage were 90%.

Virulence features, such as toxins, secretion systems, iron acquisition systems, and predicted virulence levels (pathogenicity islands and toxin clusters) were identified by screening the genome against the Virulence Factors Data Base [19] using Abricate [13]. Any coverage < 90% was excluded.

Plasmid replicon types were identified using ABRicate v1.0.1 screening against the PlasmidFinder database, applying a minimum threshold of 90% sequence identity and 90% coverage for replicon assignment. To complement replicon screening, plasmid reconstruction and contig binning were performed using MOB-suite (v3.10) via the Bactopia workflow to classify contigs into putative plasmid and chromosomal bins and to assign predicted mobility types. Putative plasmid localization of AMR genes was inferred based on 1) the co-occurrence of AMR genes and plasmid replicons on the same contig; 2) concordance of with MOB-suite plasmid bin assignments; and 3) the presence of mobilization genes. MOB-suite reconstruction relies on reference-supported clustering against curated plasmid databases, providing additional evidence for plasmid bin assignment.

Chromosomal localization of *bla*NDM-1 was inferred from its detection on contigs without identifiable plasmid replicon markers, lack of plasmid bin assignment, and its genomic context consistent with chromosomal regions. Given the inherent limitations of short-read sequencing, these findings represent *in silico* predictions of gene-plasmid associations and do not constitute definitive plasmid reconstruction. Therefore, the plasmid features and gene-plasmid associations (Tables 1, 2) represent *in silico* predictions rather than fully resolved plasmid structures.

**Table 1:** Predictive Features of Plasmid Replicons, Predicted Mobility (Based on the Presence of Relaxase Genes), Corresponding Accession Numbers, and the Closest Taxonomic Matches

Sample ID	Number of contigs	Size	Rep type(s)	Rep type accession(s)	Relaxase type(s)	Predicted mobility	Mash neighbor identification
99_S182:AA046	8	85,729	IncFIB,IncFII,rep_cluster_2272	000101__NZ_CP012345_00042,AJ851089,CP042519_00001	MOBF	Conjugative	*Leclercia adecarboxylata*
99_S182:AA002 (*bla*OXA-48)	9	76,687	IncL/M	JN626286	MOBP	Conjugative	*Klebsiella pneumoniae*
99_S182:AA624	7	48,854	-	-	-	Non-mobilizable	*Citrobacter freundii*
99_S182:AA810	1	12,614	-	-	-	Non-mobilizable	*Escherichia coli*
99_S182:AD090	1	11,161	-	-	-	Non-mobilizable	*Escherichia coli*
99_S182:novel_58d38249caaadef4bd119e14ee13c197	1	8,521	IncR	000204__CP008701_00115	-	Non-mobilizable	*Escherichia coli*
99_S182:AC292	1	8,497	IncP	000173__NC_007100_00053	MOBQ	Mobilizable	*Citrobacter sp. CF971*
99_S182:AC508 (*bla*KPC-2)	1	7,993	IncQ2	KX946994_00006	MOBP	Mobilizable	*Klebsiella oxytoca*

Plasmid replicon types were identified using ABRicate v1.0.1 screening against the PlasmidFinder database, plasmid reconstruction and contig binning were performed using MOB-suite (v3.10) via the Bactopia workflow. Underscores in identifiers reflect database-generated annotation names and were retained as reported.

**Table 2:** Description of *Bla*OXA-48 and *Bla*KPC-2 Carbapenemase Gene and Its Associated Antibiotics Resistance Profile

Start	End	Gene	Gaps	Coverage	Identity	Accession	Product	Resistance
1526	2323	*bla*OXA-48_1	0/0	100	100	AY236073	blaOXA-48	Amoxicillin, amoxicillin + clavulanic acid, ampicillin, ampicillin + clavulanic acid, imipenem, meropenem, piperacillin, piperacillin + tazobactam
360	1241	*bla*KPC-2_1	0/0	100	100	AY034847	blaKPC-2	Amoxicillin, amoxicillin + clavulanic acid, ampicillin, ampicillin + clavulanic acid, aztreonam, cefepime, cefotaxime, cefoxitin, ceftazidime, ertapenem, imipenem, meropenem, piperacillin, piperacillin + tazobactam, ticarcillin, ticarcillin + clavulanic acid

The table shows *in silico* prediction of the genomic location of the genes, sequence identity, and coverage (100%). Assembled contigs were screened against curated antimicrobial resistance databases (ResFinder/CARD) using sequence alignment tools (BLAST) with a defined threshold coverage (≥ 90%). BlaOXA-48 is a class D carbapenemase that is often associated with mild resistance to carbapenems and difficult to detect phenotypically, whereas blaKPC-2 is a class A carbapenemase that often demonstrates a high level of resistance to all β-lactam agents.

### Results

#### Sequencing quality control

The sequencing run achieved a Q30 score of 88%, indicating high base-calling accuracy. Assembly quality metrics for the sample included an N50 contig length of 111,647 bp, an L50 of 14, and a sequencing depth of coverage of 99.38 ×, indicating robust genome representation.

The draft assembly, using the St. Petersburg genome *de novo* assembler (SPAdes), yielded 129 contigs, with a total genome size of 5,287,670 bp and a GC content of 53.34%. Functional annotation with Prokka identified 5,112 genomic features, including 5,073 coding sequences, two rRNA genes, 36 tRNA genes, and 855 hypothetical proteins, consistent with *Citrobacter* spp. genome.

Taxonomic classification was performed using similarity- and phylogeny-based methods. Mash analysis against the NCBI Reference Sequence (RefSeq) database identified *Citrobacter* species NLAE-zl-C269 (NCBI:txid 1855329) as the closest reference genome, with a Mash identity of 99.09% and 826 of 1,000 shared hashes. MLST analysis using the *C. freundii* scheme assigned the isolate to ST714. However, phylogenomic analysis using Sourmash and the GTDB conclusively identified the isolate as *C. amalonaticus*. These combined results confirmed the identity of the isolate as a member of the *C. amalonaticus* complex, which is similar to a known *Citrobacter* strain and has consistent phylogenetic placement. Given the superior resolution of genome-wide analysis and phylogenomic placement over MLST, we assigned the isolate to *C. amalonaticus* as per GTDB.

AMR profiling detected β-lactamase genes including *bla*KPC-2, *bla*NDM-1, *bla*OXA-48, each known to confer resistance to broad-spectrum and carbapenem antibiotics. Additional resistance determinants included *aac(6′)-Ib* for aminoglycosides, *mph(A)* for macrolides, *qnrVC4* and *oqxB* for quinolones, *cmlA5* for phenicols, and *ble* for bleomycin resistance. All genes were reported to have high nucleotide identity (≥ 96.1%) and full or near-full coverage, indicating their potential presence and functional relevance (Tables 1–3).

**Table 3:** Antimicrobial Resistance Markers Predicted to Be Chromosomally Encoded (Identified Using ResFinder, CARD, and ARG-ANNOT)

Contig	Start	End	Gene	Gaps	Coverage	Identity	Accession	Product	Resistance
99_S182_00081	320	1132	blaNDM-1_1	0/0	100	100	FN396876	blaNDM-1	Amoxicillin, amoxicillin + clavulanic acid, ampicillin, ampicillin + clavulanic acid, cefepime, cefixime, cefotaxime, cefoxitin, ceftazidime, ertapenem, imipenem, meropenem, piperacillin, piperacillin + tazobactam
99_S182_00063	2782	4041	cmlA1_1	0/0	100	100	M64556	cmlA1	Chloramphenicol
99_S182_00063	11531	12452	mph(A)_2	1/1	100	100	U36578	mph(A)	Erythromycin, azithromycin, spiramycin, telithromycin
99_S182_00063	4977	5633	qnrVC4_1	0/0	100	100	GQ891757	qnrVC4	Ciprofloxacin

The stress-resistance gene profile included multiple genes associated with tolerance to heavy metals, acids, and biocidal agents, such as *ars (A–D)*, *merR*, *merE, merA*, *merD*, *merP*, *merT*, *merC* (mercury resistance), *pcoA-E*, *pcoR*, *pcoS*, *silP*, *silB silA*, *silC*, *silF*, *silE* (copper and silver resistance), in addition to *qacE* gene, which provides resistance to quaternary ammonium compound-based disinfectants. *In silico* genomic analysis suggested the presence of eight plasmid replicons, as well as integrons, transposons, and insertion sequences. Computational analysis of plasmid-associated carbapenemase genes indicated that *bla*NDM-1 was encoded chromosomally, whereas *bla*KPC-2 and *bla*OXA-48 appeared to be associated with the IncQ2- and IncL/M-type replicons, respectively. The high sequence similarity between these replicons and known plasmids from *Klebsiella pneumoniae* and *K. oxytoca* suggests possible horizontal gene transfer events. However, due to short-read sequencing limitations, the complete plasmid architecture, structural validation, and definitive localization of resistance genes within mobile genetic elements cannot be fully resolved. These findings should be interpreted as genomic predictions rather than as definitive plasmid reconstructions.

#### Nucleotide sequence accession number

The complete sequence of the strain was submitted to GenBank for a BioProject number PRJNA1284363.

## Discussion

*C. amalonaticus* is rarely encountered in clinical infections, and its association with triple carbapenemase production has major clinical implications because carbapenemases are not the most common type of β-lactamases found in *Citrobacter* spp. Although most *Citrobacter*-related case studies focus on *C. freundii*, since it is a frequently isolated species, few studies have described the occurrence of MDR *C. amalonaticus*. For example, several reports have described the presence of multiple AMR genes in *C. freundii*, such as those encoding NDM, KPC, VIM, OXA-181 and OXA-48, in addition to other narrow spectrum β-lactamase-producing genes, such as the intrinsic cephalosporinase AmpC [20, 21].

In an extensive surveillance study that included 769 CRE, Cabello et al identified five isolates of *C. freundii* and one isolate of *C. amalonaticus* carrying KPC-3 and OXA-48 on different plasmid [22]. *In silico* analysis of our case suggested the possible occurrence of multiple plasmid replicons, of which two (IncQ2 and IncL/M) were computationally associated with the carbapenemase genes *bla*KPC-2 and *bla*OXA-48, respectively. Replicon typing and sequence similarity to known *Klebsiella* plasmids indicated their potential for horizontal gene transfer (Tables 1–3). The widespread occurrence of horizontal gene transfer remains one of the biggest challenges in controlling drug resistance [23].

In the present study, the presence of multiple carbapenem-inactivating genes was detected in *C. amalonaticus*. Upon reviewing the patient’s medical records, we noticed the development of this infection after long-term use of meropenem, which may suggest selection pressure associated with the prolonged use of carbapenems [24].

The pan-resistance of the isolate, including ceftazidime-avibactam resistance, underscores a critical therapeutic challenge. This resistance is likely due to the combined activity of the metallo-β-lactamase NDM-1 (not inhibited by avibactam) and the serine carbapenemases KPC-2 and OXA-48. This leaves only a few viable options, with *in vitro* activity retained only for amikacin, fosfomycin, nitrofurantoin, and colistin [25]. The potential presence of plasmid-borne *bla*KPC-2 and *bla*OXA-48 in this isolate raises serious concerns regarding infection control. Plasmids carrying carbapenemase genes facilitate horizontal gene transfer across the *Enterobacterales*, thereby accelerating the spread of resistance in healthcare environments. This risk is amplified in hospital settings, where selective pressure and close patient contact favor plasmid dissemination. Therefore, enhanced surveillance, strict contact precaution, and plasmid epidemiology monitoring are warranted to prevent outbreaks and limit further therapeutic compromises. In situations with limited isolation capacity, such isolates should be prioritized for strict contact isolation to prevent onward transmission [22].

To our knowledge, this is the first documented clinical *C. amalonaticus* isolate co-harboring three carbapenemase genes (*bla*NDM-1, *bla*KPC-2, and *bla*OXA-48) in Saudi Arabia. Although *C. amalonaticus* has been reported in local studies that evaluated the prevalence of hospital-acquired infections, no previous reports have described isolates carrying triple carbapenemase genes [26]. While our analysis identified the chromosomal localization of *bla*NDM-1, detailed characterization of the surrounding genetic context, including insertion sequences (e.g., ISAba125), transposons, and integrative elements, was not performed because of assembly fragmentation inherent to short-read sequencing. Chromosomal integration of carbapenemase genes is clinically significant, as it may confer greater genetic stability than plasmid carriage, potentially facilitating vertical transmission and persistence within bacterial populations [27]. The mechanism of chromosomal acquisition (e.g., plasmid-to-chromosome transfer, integrative conjugative elements, or transposon-mediated insertion) remains unresolved and warrants further investigation using long-read sequencing technologies.

This case has significant implications for AMR surveillance in Saudi Arabia and the broader Gulf region. Although carbapenem resistance in *Enterobacterales* has been documented regionally with pooled prevalence rates of 6.6–18.6% [3], carbapenemase production in *Citrobacter* spp. remains underreported. The convergence of three carbapenemase genes in a single isolate represents an alarming escalation in the complexity of resistance within the region. Given the country’s role as a hub for international healthcare and the annual Hajj season, which involves millions of visitors, the detection of such high-risk isolates underscores the need for enhanced genomic surveillance, rapid molecular diagnostics, and stringent infection prevention protocols to prevent local dissemination and cross-border transmission. This case highlights that carbapenem resistance in Saudi Arabia is no longer limited to well-recognized high-risk pathogens (*Klebsiella pneumoniae*, *Escherichia coli*) but is emerging in opportunistic genera such as *Citrobacter*, necessitating broadened surveillance strategies.

This case report has several limitations that warrant further investigation. Although the chromosomal localization of *bla*NDM-1 was identified, detailed analysis of the surrounding insertion sequences, transposons, or integrative elements was not performed; consequently, the genetic mechanism underlying chromosomal integration remains unresolved. Additionally, although the short-read sequencing approach enables comprehensive resistance profiling and robust taxonomic classification, it has inherent limitations for complete plasmid reconstruction and mobile genetic element characterization, which typically require long-read or hybrid sequencing strategies. With an average fragment size of approximately 600 bp, repetitive regions and structurally complex loci cannot be fully resolved. Genomic resolution is insufficient to definitively determine plasmid architecture, achieve complete plasmid circularization, or assign AMR genes unambiguously to plasmids versus chromosomes. Plasmid-related and localization findings should therefore be interpreted as computationally inferred rather than experimentally confirmed and are best regarded as suggestive rather than definitive.

### Conclusions

Genomic characterization of this isolate revealed the co-occurrence of three high-risk carbapenemase genes (*bla*NDM-1, *bla*KPC-2, *bla*OXA-48) in a single *C. amalonaticus* strain. *In silico* analysis suggested that some of these resistance determinants were associated with plasmid replicons, highlighting the potential for horizontal gene transfer within *Enterobacterales* populations. This case highlights the urgent escalation in the epidemiology of carbapenem resistance in Saudi Arabia and underscores that such resistance is now emerging in opportunistic genera beyond classical high-risk pathogens. Collectively, these findings reinforce the need for enhanced genomic surveillance, rapid molecular diagnostics, and stringent infection prevention and control measures to prevent the dissemination of high-risk isolates.

### Learning points

Carbapenem-resistant *C. amalonaticus*, whilst less frequently encountered than *Klebsiella pneumoniae* or *Escherichia coli*, is capable of accumulating multiple carbapenemase genes and should not be underestimated as a clinical and epidemiological threat.

Standard MIC-based susceptibility reporting does not capture the mechanistic complexity of multi-carbapenemase co-carriage; WGS is essential for characterizing resistance architecture and informing targeted infection control.

*In vitro* susceptibility to newer agents e.g., ceftazidime-avibactam may be rendered clinically irrelevant by the concurrent expression of multiple carbapenemases that are not inhibited by the drug.

Short-read WGS provides high-resolution resistance gene cataloguing but cannot reliably reconstruct complete plasmid topology; long-read or hybrid sequencing is required for definitive mobile genetic element characterization.

In settings with high migrant worker populations, as in the case of Gulf Cooperation Council (GCC), enhanced surveillance for carbapenemase-producing organisms in surgical and wound-care settings is warranted.

## Data Availability

The data supporting the findings of this study are available from the corresponding author upon reasonable request.

## Abbreviations

CP: carbapenemase-producing
SSI: surgical site infection
WGS: whole-genome sequencing
GTDB: Genome Taxonomy Database
CRE: carbapenem resistant *Enterobacterales*
